# Synthesis, Reactions and Antimicrobial Activities of 8-Ethoxycoumarin Derivatives

**DOI:** 10.3390/molecules17010971

**Published:** 2012-01-18

**Authors:** Hany M. Mohamed, Ashraf H. F. Abd El-Wahab, Kamal A. Ahmed, Ahmed M. El-Agrody, Ahmed H. Bedair, Fathy A. Eid, Mostafa M. Khafagy

**Affiliations:** 1 Chemistry Department, Faculty of Medicine, Jazan University, Jazan 82621, Saudi Arabia; 2 Chemistry Department, Faculty of Science, Jazan University, Jazan 2097, Saudi Arabia; 3 Chemistry Department, Faculty of Science, Al-Azhar University, Nasr City, Cairo 11884, Egypt; 4 Chemistry Department, Faculty of Science, King Khalid University, Abha 9004, Saudi Arabia

**Keywords:** 3-acetyl-8-ethoxycoumarin, bromination, active methylene, thiazole derivatives, thiosemicarbazide, KSCN, antimicrobial activities

## Abstract

Condensation of 3-acetyl-8-ethoxycoumarin (**3**) with thiosemicarbazide gave ethylidenehydrazinecarbothioamide **5**, which was transformed into the thiazolidin-4-one derivatives **6**,**7**. Interaction of **3** with DMF/POCl_3_ gave *β*-chloroacroline derivative **8**. Treatment of **3** with malononitrile gave benzo[*c*]chromone and 2-aminobenzonitrile derivatives **9** and **10**, respectively with respect to the reaction conditions. Condensation of 3-(2-bromoacetyl)-8-ethoxycoumarin (**4**) with *o*-phenylenediamine gave 3-(quioxaline-2-yl)-8-ethoxycoumarin hydrobromide (**11**), while **4** reacted with 2-aminopyridine to give chromenopyridopyrimidine derivative **12**. Condensation of **4** with potassium thio-cyanate/methanol gave an unexpected derivative, 2*H*-chromeno-3-carboxy(methyl-carbonimidic)thioanhydride **16**, which upon treatment with (NH_2_)_2_·H_2_O gave 3-ethoxy-2-hydroxybenzaldehyde azine **19**. Interaction of **4** with thiourea derivatives gave thiazole derivatives **20a–c**. The structures of the newly synthesized compounds were confirmed by their spectra data. The newly synthesized compounds were also screened for their antimicrobial activity.

## 1. Introduction

Coumarin and its derivatives are used as additives in food, perfumes, cosmetics, pharmaceuticals, agrochemicals [1,2], for their spasmolytic, cardiothioc, antiviral, anticancer properties [3,4] and as laser dyes in the blue-green region. These types of dyes have been employed as labels for fluorescent energy transfer experiments [5,6]. Coumarin compounds also form a group of more than 40 drugs, which are widely used in medicine as anticoagulant, hypertensive, antiarrhythmic and immunomodulant agents [7]. Many coumarins were tested for various kinds of biological activity and their structures established based on chemical analytical techniques and spectroscopic methods [8,9,10,11,12,13,14,15]. The present study is a part of our programme [16,17,18,19,20,21,22,23,24,25,26,27,28] directed towards the synthesis of novel coumarin and chromene derivatives and evaluation of their antimicrobial activities. 

## 2. Results and Discussion

### 2.1. Chemistry

**Scheme 1:**
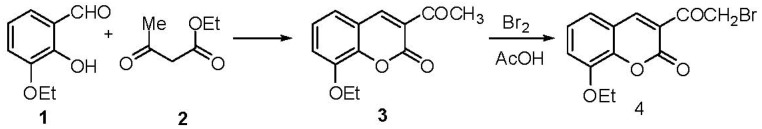
Synthesis of 3-(2-bromoacetyl)-8-ethoxycoumarin (**4**).

Treatment of 3-ethoxysalicylaldehyde (**1**) with ethyl acetoacetate (**2**) in boiling ethanol containing few drops of piperidine afforded 3-acetyl-8-ethoxycoumarin (**3**) [29]. Bromination of **3** in acetic acidgave the corresponding *ω*-bromo-8-ethoxy-3-acetylcoumarin (**4**) [29] (Scheme 1).

**Scheme 2:**
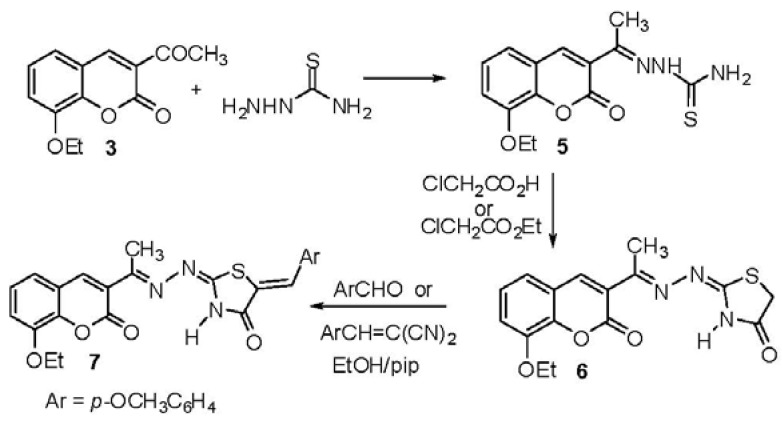
Synthesis hydrazone derivatives **5–7**.

Condensation of **3** with thiosemicarbazide afforded 2-[1-(8-ethoxycoumarin-3-yl)ethylidene]-hydrazinecarbothioamide (**5**). Reaction of **5** with chloroacetic acid or ethyl chloroacetate afforded 2-[(1-(8-ethoxycoumarin-3-yl)ethylidene]hydrazonothiazolidin-4-one (**6**). Treatment of the thiazolidin-4-one derivative **6** with *p*-methoxybenzaldehyde or *α*-cyano-*p*-methoxycinnamonitrile in ethanolic piperidine afforded the corresponding 2-[(1-(8-ethoxycoumarin-3-yl)ethylidene)hydrazono]-5-(4-methoxybenzylidene)thiazolidin-4-one (**7**) (Scheme 2).

Structures **5–7** were established on the basis of spectral data. IR spectra showed the presence of NH_2_ and NH absorptions at υ 3,404, 3,254, 3,174 cm^−1^ and CO at υ 1,720 cm^−1^ for **5**, NH at υ 3,147 cm^−1^, υ 2,977 C-H (aliphatic), CO at υ 1,720, 1,648 cm^−1^ for **6** and a CO at υ 1,705, 1,655 cm^−1^ and υ 1,605 cm^−1^ for N=C and 2,939.3 cm^−1^ for **7**. The ^1^H-NMR spectrum of **6** showed signals at δ 12.05 ppm (brs, 1H, NH), 3.87 ppm (s, 2H, CH_2_), 2.49 ppm (s, 3H, CH_3_) and that of **7** signals atδ 12.40 (brs, 1H, NH), 8.44 ppm (s, 1H, 4-H), 8.25 ppm (s, 1H, =CH-Ar), 3.84 ppm (s, 3H, OCH_3_). The mass spectra of compounds **5**,**6** provided additional evidence in support of the proposed structures.

**Scheme 3:**
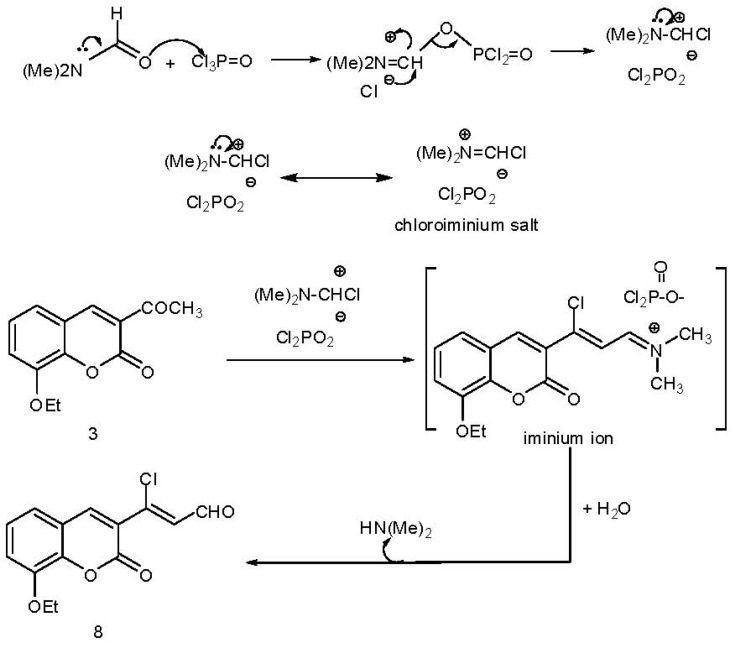
Synthesis of *β*-chloroacroline derivative **8**.

Treatment of 8-ethoxy-3-acetylcoumarin (**3**) with DMF/POCl_3_ gave 3-chloro-3-(8-ethoxycoumarin-3-yl)acrylaldehyde(**8**) (Scheme 3). The formation of **8** indicates that the enolate form of **3** attacks the chloroiminium salt to gave the iminium ion which is hydrolyzed to the *β*-chloroacroline derivative **8** [30].

**Scheme 4:**
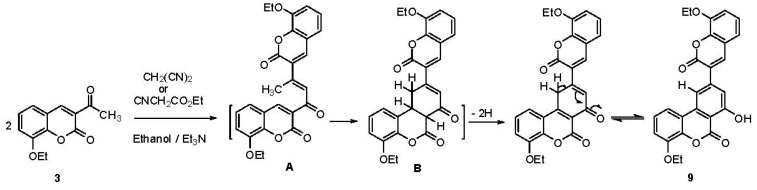
Synthesis of benzo[*c*]chromene-6-one derivative **9**.

Interaction of **3** with malononitrile or ethyl cyanoacetate in ethanolic triethylamine solution gave the same product, the benzo[*c*]chromene-6-one derivative **9** (Scheme 4).

The formation of **9** can be explained by a self condensation of **3** to give a chalcone (**A**) as intermediate which undergoes an intramolecular cyclization through the addition of an active methylene group to the activated 3,4-double bond forming the intermediate (**B**), which then underges spontaneous oxidation to the final product **9** [31] (Scheme 4).

**Scheme 5:**
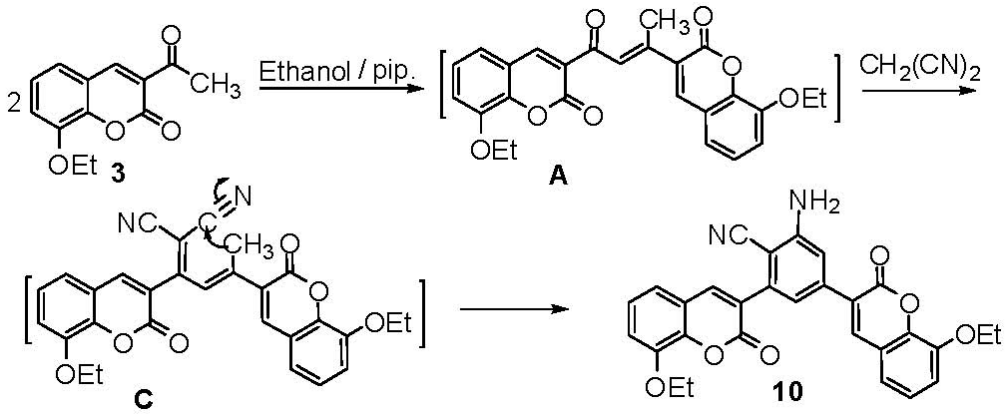
Synthesis of 2-amino-4,6-bis(8-ethoxycouarin-3-yl)benzonitrile (**10**).

Treatment of **3** with malononitrile in boiling methanolic piperidine solution instead of triethyl-amine, gave a product which formulated as 2-amino-4,6-bis(8-ethoxycouarin-3-yl)benzonitrile (**10**) (Scheme 5). The formation of **10** could be explained by condensation of the intermediate (**A**) with malononitrile to give another intermediate (**C**) which underges intramolecular cyclization through the nucleophilc addition of active methylene group to one of the carbonitrile functions followed by aromatization [32] (Scheme 5).

**Scheme 6:**
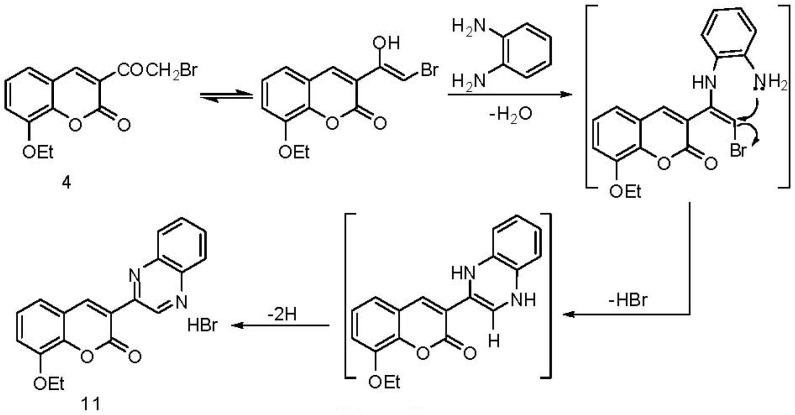
Synthesis of 3-(quinoxalin-2-yl)-8-ethoxycoumarin hydrobromide (**11**).

Treatment of **4** with an equimolar amount of *o*-phenylenediamine in boiling methanol resulted in the formation of 3-(quinoxalin-2-yl)-8-ethoxycoumarin hydrobromide (**11**) (Scheme 6). The formation of **11** [33] may be explained by cyclocondensation of *ω*-bromo-8-ethoxy-3-acetylcoumarin (**4**) with *o*-phenylenediamine, followed by subsequent oxidation (Scheme 6).

**Scheme 7:**
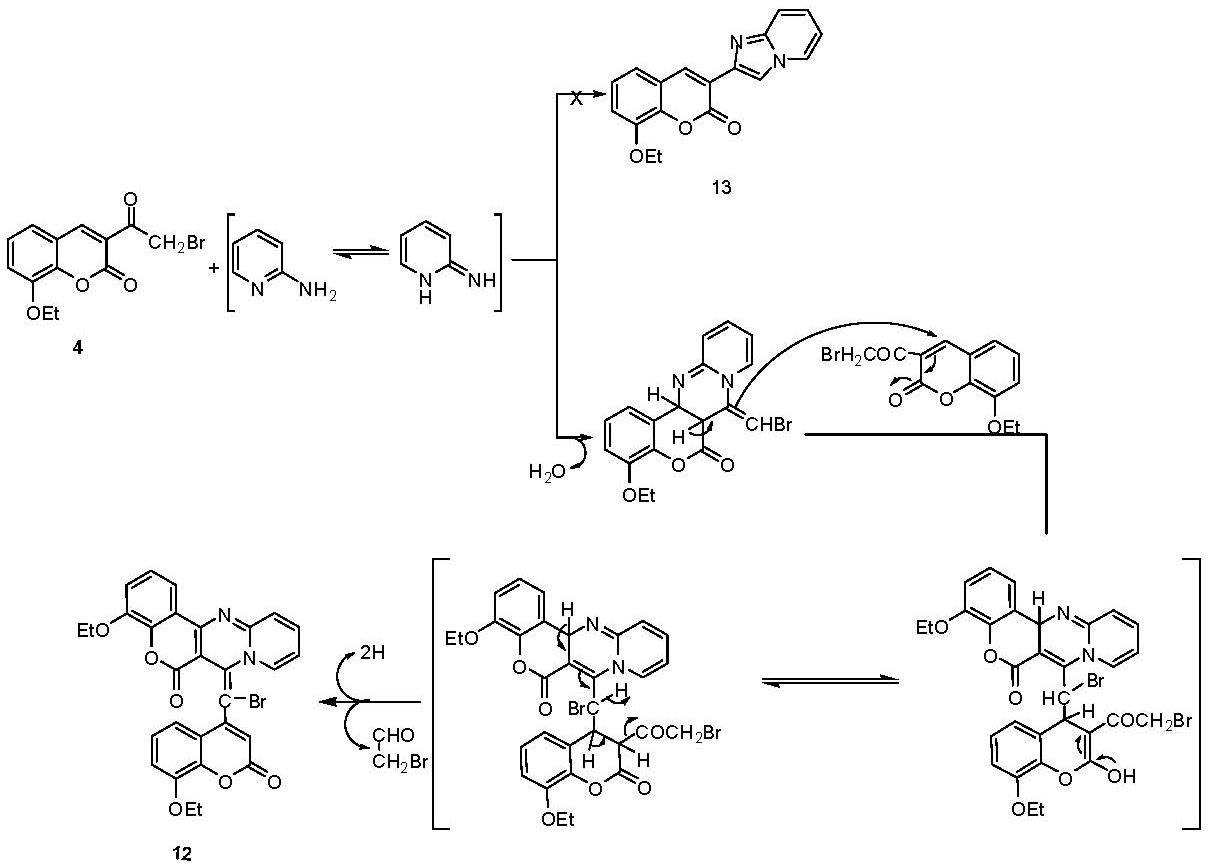
Synthesis of chromenopyridopyrimidine **12**.

In a similar manner, **4** reacted with 2-aminopyridine to give the 4-ethoxychromeno[4,3-*d*]pyrido[1,2-*a*]pyrimidin-6-(7*H*)-one derivative **12** rather than the expected 3-imidazo[1,2-*a*]pyridine-2-yl-8-ethoxychrome-2-one **13** [34] (Scheme 7).

**Scheme 8:**
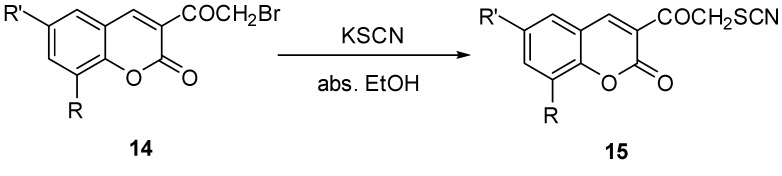
Synthesis of 3-thiocyanato derivatives **15**.

The formation of **12** can be explained by a nucleophilic addition of the more nucleophilic cyclic secondary nitrogen of 2-aminopyridine to the electrophilic carbonyl carbon of 3-(2-bromoacetyl)-8-ethoxycoumarin (**4**) instead of nucleophilic replacement of bromine; the addition intermediate undergoes a subsequent cyclodehydration with the addition of the exonucleophilic nitrogen atom of 2-aminopyridine at position 2 to the active site of the coumarin ring (C-4), and the intermediate then acts as a nucleophile which attacks a second molecule of **4** at C-4 followed by aromatization and elimination of a bromoacetaldehyde molecule to give the final product **12**. Previously Ramanna *et al*. [35] had stated that 3-(*ω*-bromoacetyl)coumarins **14** on reaction with potassium thiocyanate gave 3-thiocyanatoacetylcoumarins **15** (Scheme 8).

**Scheme 9:**
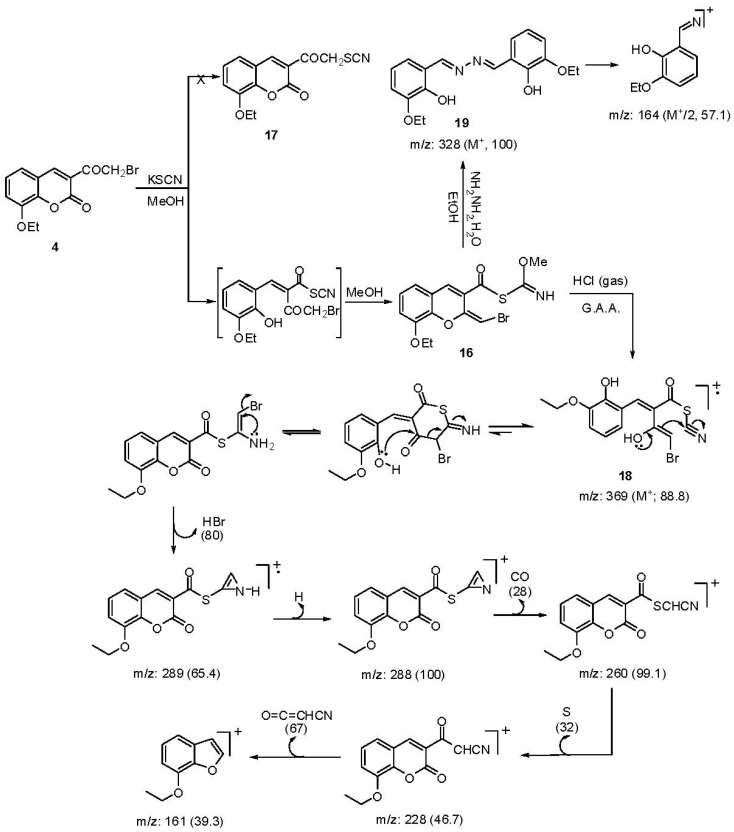
Synthesis of compounds **16**, **18** and **19** with MS fragmentaion patterns.

In the present research, treatment of **4** with KSCN in boiling methanol gave 2-bromoethylene-8-ethoxy-2H-chromene-3-carboxylic(methylcarbonimidic)thioanhydride (**16**) rather than the corresponding 3-thiocyanatoacetylcoumarin derivative **17 **(Scheme 9). 

The structure was supported by the following evidence: (i) in the IR spectrum, the absence of SCN and the presence of NH at 3,141 cm^−1^; (ii) the ^1^H-NMR spectrum exhibited a characteristic singlet for OCH_3_ protons at 4.10 ppm and at 7.88 as singlet signal for =CH-Br; (iii) the mass spectrum gave a molecular ion peak at *m/z* (%): 385 (M^+^+2, 37.3), 383 (M^+^, 42.4) with a base peak at 303 (M-HBr, 100). The formation of **16** indicates that the strong nucleophile SCN^−^ attacks the lactone carbonyl with concomitant addition of a methanol molecule and cyclization to give a chromene nucleus. When **16** was treated with HCl_gas_/AcOH, a methanol molecule was eliminated and the chromene ring was opened to furnish 4-bromo-2-(3-ethoxy-2-hydroxybenzylidene)-3-hydroxybut-3-enoic cyanic thioanhydride (**18**) (Scheme 9). The mass spectrum of **18** gave a molecular ion peak at *m/z* (%): 369 (M+, 88.8) together with a base peak at 288 (100), and other peaks at 289 (65.4), 260 (99.0), 228 (46.7), 161 (39.3). Interaction of **16** with ethanolic hydrazine hydrate solution effected ring opening with scission of the C_3_-C_4_ bond [36] and gave 3-ethoxy-2-hydroxybenzaldehyde azine (**19**) (Scheme 9). IR gave υ C=N at 1,562 and 1,597 cm^−1^, and OH at 3,302 cm^−1^, ^1^H-NMR ppm: 9.20 (s, 1H, CH=N), 12.10, 12.45 (phenolic OH). Its mass spectrum gave a molecular ion peak at *m/z* (intensity %): 328 (100) as a base peak, and other peaks at 164 (M/2, 57.12), 136 (45.7), 121 (50.9), 80 (65.3).

**Scheme 10:**
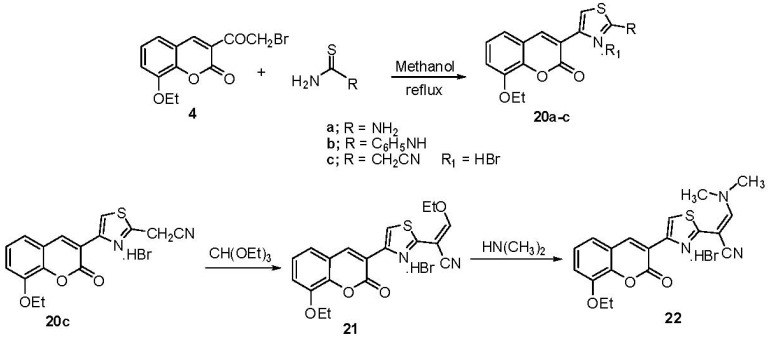
Synthesis of coumarinothiazole derivatives **20**, **21** and **22**.

Interaction of **4** with thiourea, phenylthiourea and cyanothioacetamide in boiling methanol afforded the thiazole derivatives **20a–c** [37,38,39] respectively (Scheme 10). Interaction of **20c** with neat triethyl orthoformate under reflux give the ethoxymethyleneamino derivative **21**, which reacted with dimethylamine to give *N*, *N*-dimethylaminoethyleneamino derivative **22** (Scheme 10).

The structures of **20–22** were confirmed by their spectral data. The IR spectra showed NH_2_ bands at υ 3,387, 3,302, 3,148 cm^−1^ and a CO band at υ 1,705 cm^−1^ for **20a**; NH bands at υ 3,302, 3,148 cm^−1^ and CO band at υ 1,697 cm^−1^ for **20b**, and CN band at υ 2,262 cm^−1^ and CO band at υ 1,722 cm^−1^ for **20c**; C-H (aliphatic) at υ 2,978 cm^−1^, CN band at υ 2,222 cm^−1^ a CO band at υ 1,728 cm^−1^ for **21** and a CN band at υ 2,191 cm^−1^, a CO band at υ 1,720 cm^−1^, C-H (aliphatic) at υ 2,927 & 2,874 cm^−1^ for **22**. Characteristic ^1^H-NMR resonances were observed at δ 10.31 (s, 1H, HNPh) and 8.61 ppm (s, 1H, H-4) for **20b**, δ 8.42 (s, 1H, 4-H) and 4.55 ppm (s, 2H, CH_2_) 8.71 (s, 1H, thiazole-H) for **20c**, δ 8.43 (s, 1H, 4-H) 8.15 (s, 1H, =CHOEt), 8.70 (s, 1H, thiazole-H) for **21** and 8.12 ppm (s, 1H, 4-H) and 3.28 ppm (s, 6H, N(CH_3_)_2_), 7.89 [s, 1H, =CHN(Me)_2_], 8.74 (s, 1H, thiazole-H) for **22**. The mass spectrum of **20c** gave a molecular ion peak at *m/z* (%) 394 (M^+^+2, 24.90) and 392 (M^+^, 28.90) with a base peak at 284 (M^+^-(HBr+CO), 100); for **21**: *m/z* (%) 448 (M^+^, 12.1), 450 (M^+^+2, 10.1) and a base peak at 368 (M-HBr, 100); for **22**: *m/z*: 449 (M^+^+2, 14.1), 447 (M^+^, 18), 367 (M^+^-HBr, 100).

**Scheme 11:**
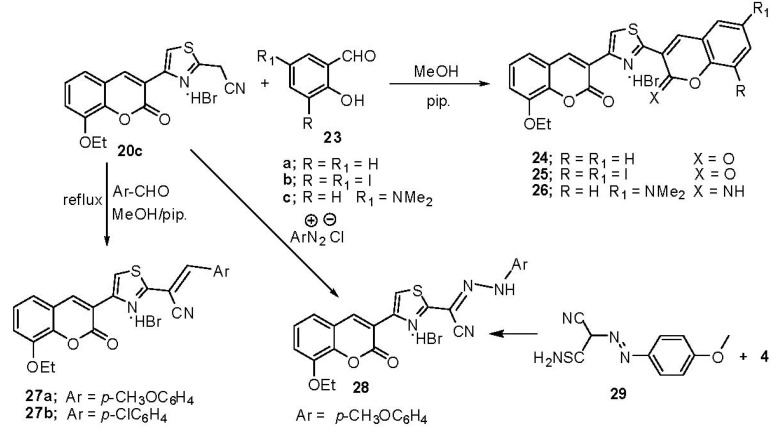
Synthesis of coumarin-3-ylthiazole derivatives **24–28**.

Condensation of **20c** with various aromatic aldehydes **23** afforded the corresponding dicoumarin-3-ylthiazole derivatives **24**, **25** and iminocoumarin derivative **26**, while condensation of **20c** with aromatic aldehydes in methanolic piperidine solution give **27a**,**b**. Compound **20c** was also readily coupled with *p*-methoxybenzenediazonium chloride to afford **28** (Scheme 11). The structure of **28** was supported by its independent synthesis from **4** and (4-methoxyphenylazo)-2-cyanoethanethioamide (**29**) (Scheme 11).

Structures **24–28** were established on the basis of spectral data. The IR spectra showed the presence of a CO bands at υ 1,720 cm^−1^ for **24–26**, while a NH band appeared at υ 3,449, 3,209 cm^−1^ for **26**; a CN and at υ 2,207 cm^−1^ a CO band at υ 1,728 cm^−1^ for **27a**; a CN band at υ 2,214 cm^−1^ and a CO band at υ 1,720 cm^−1^ for **27b** and a NH band at υ 3,156 cm^−1^ a CN band at υ 2,214 cm^−1^ CO at υ 1,736 cm^−1^ for **28**. The ^1^H-NMR spectra showed two signals at δ 8.95, 8.54 ppm (2s, 2H, H-4) 9.14 (s, 1H, thiazole-H) for **24**; at δ 8.39 (s, 2H, 2H-4) and 3.31 ppm (s, 6H, 2CH_3_) for **26 **and δ 11.93 (s, 1H, NH, exchangeable with D_2_O), 8.39 (s, 1H, H-4) and 3.76 ppm (s, 3H, OCH_3_) for **28**. The mass spectra of compounds **24–28** provided additional evidence for the proposed structures.

## 3. Experimental

### 3.1. General

Melting points were determined with a Stuart Scientific Co. Ltd. apparatus. IR spectra were determined as KBr pellets on a Jasco FT/IR 5300 spectrophotometer. ^1^H-NMR spectra were recorded using a Varian Mercury (300 MHz) spectrometer. The mass spectra recorded on a Shimadzu GC-MS QP 1000 EX spectrometer. Elemental analyses were performed on a Perkin-Elmer 240 microanalyser in the Cairo University Faculty of Science.

*Synthesis of 3-acetyl-8-ethoxycoumarin* (**3**). 3-Ethoxysalicylaldehyde **1** (10 mmol) was refluxed with ethyl acetoacetate **2** (10 mmol) in ethanolic/piperidine solution (20 mL, 0.5 mL) for two hours. The precipitate formed on cooling was collected by filtration, washed with cold ethanol and dried. The solid formed was recrystallized from diluted ethanol to give pale yellow crystals; yield 88%; m.p. 130–132 °C, lit. (135–7 °C) [29]; IR (KBr) υ (cm^−1^): 3,060, 2,976, 2,874 (CH stretching), 1,730, 1,678 (CO); ^1^H-NMR (DMSO-d_6_) δ: 1.40 (t, 3H, CH_3_, *J* = 7.2 Hz), 2.58 (s, 3H, COCH_3_), 4.19 (q, 2H, CH_2_, *J* = 7.2 Hz), 7.31-7.65 (m, 3H, Ar-H), 8.59 (s, 1H, H-4); ^13^C-NMR (DMSO-d_6_) δ: 13.90 (CH_3_-ester), 25.72 (CH_3_-acetyl), 62.33 (CH_2_-ester), 115.40 (C-7), 118.21 (C-5), 120.11 (C-4a), 126.37 (C-6), 128.00 (C-3), 145.03 (C-4), 150.80 (C-8a), 154.12 (C-8), 160.04 (CO-lactone), 175.19 (CO-acetyl); Anal. calcd. for C_13_H_12_O_4_: C, 67.22; H 5.17; found: C, 67.20; H, 5.16%.

*Synthesis of 3-(2-bromoacetyl)-8-ethoxycoumarin* (**4**). A solution of **3** (10 mmol) in acetic acid (10 mL) was stirred with bromine (0.50 mL, 10 mmol) for 2 hours in direct sun-light. The solid formed was collected by filtration, washed with acetic acid, then ethanol, and dried. The product was recrystallized from ethanol to give yellow crystals; yield: 79%; m.p. 180–182 °C; IR (KBr) υ (cm^−1^): 3,080, 2,972, 2,952, (CH stretching), 1,722, 1,692 (CO); ^1^H-NMR (CDCl_3_) δ: 1.54 (t, 3H, CH_3_, *J* = 7.2 Hz), 4.23 (q, 2H, CH_2_, *J* = 7.2 Hz), 4.77 (s, 2H, CH_2_), 7.19–7.32 (m, 3H, Ar-H), 8.60 (s, 1H, H-4); ^13^C-NMR (DMSO-d_6_) δ: 13.73 (CH_3_-ester), 35.10 (CH_2_Br) 62.13 (CH_2_-ester), 115.47 (C-7), 118.43 (C-5), 120.01 (C-4a), 125.75 (C-6), 127.92 (C-3), 145.53 (C-4), 151.42 (C-8a), 154.19 (C-8), 162.70 (CO-lactone), 173.03 (CO-acetyl); MS *m/z* (%): 312 (M^+^+2, 29.02), 310 (M^+^, 29.29), 231 (70.11), 217 (58.15), 203 (61.9), 189 (100); Anal. calcd. for C_13_H_11_BrO_4_: C, 50.32; H 3.55; found: C, 50.33; H, 3.57%.

*Synthesis of 2-(1-(8-ethoxycoumarin-3-yl)ethylidene)hydrazinecarbothioamide* (**5**). A solution of **3** (10 mmol) in DMF (10 mL) was refluxed with thiosemicarbazide (10 mmol) for 2 hours. The product formed was filtered off, washed with ethanol, dried and recrystallized from ethanol to give **5** as colourless needles; yield 81%; m.p. 218–220 °C,; IR (KBr) υ (cm^−1^): 3,404, 3,254, 3,174, (NH and NH_2_), 2,976 (C-H aliphatic), 1,720 (CO); MS *m/z* (%): 305 (M^+^, 42), 290 (100), 245 (23), 230 (27), 161 (24); Anal. calcd. for C_14_H_15_N_3_O_3_S: C, 55.07; H, 4.95; N, 13.76; found: C, 55.09; H, 4.94; N, 13.78%.

*Synthesis of 2-((1-(8-ethoxycoumarin-3-yl)ethylidene)hydrazono)thiazolidin-4-one* (**6**). A mixture of **5** (10 mmol) and chloroacetic acid or ethyl chloroacetate (10 mmol) was refluxed in acetic acid (20 mL) for 3 hours. The solid product was filtered off, washed with excess ethanol and recrystallized from acetic acid to give pale yellow crystals; yield 83%; m.p. 238–240 °C; IR (KBr) υ (cm^−1^): 3,147 (NH), 2,977, (CH aliphatic), 1,720, 1,648 (CO); ^1^H-NMR (DMSO-d_6_) δ: 1.41 (t, 3H, CH_3_, *J* = 6.9 Hz), 2.49 (s, 3H, CH_3_), 3.83 (s, 3H, OCH_3_), 3.87 (s, 2H, CH_2_), 4.18 (q, 2H, CH_2_, *J* = 6.9 Hz), 7.29–7.36 (m, 3H, Ar-H), 8.46 (s, 1H, H-4), 12.05 (brs, 1H, NH); MS *m/z* (%): 345 (M^+^, 100), 344 (94), 330 (58), 301 (53), 288 (61), 188 (55), 185 (51), 130 (50), 87 (59); Anal. calcd. For C_16_H_15_N_3_O_4_S: C, 55.64; H, 4.38; N, 12.17; found: C, 55.66; H, 4.37; N, 12.19%.

*Synthesis of 2-((1-(8-ethoxycoumarin-3-yl)ethylidene)hydrazono)-5-(4-methoxybenz-ylidene)thiazolidin-4-one* (**7**). A solution of **6** (5 mmol), *p*-methoxybenzaldehyde or *α*-cyano-*p*-methoxycinnamonitrile (5 mmol) in ethanol (30 mL) containing few drops of piperidine was heated under reflux for 3 hours. The solid obtained on cooling was filtered off, washed with ethanol, dried and recrystallized from acetic acid to give pale brown crystals; yield 92%; m.p. 220–2 °C; IR (KBr) υ (cm^−1^): 2,939.3 (CH aliphatic), 1,705, 1,655 (CO), 1,605 (N=C); ^1^H-NMR (DMSO-d_6_) δ: 1.42 (t, 3H, CH_3_, *J* = 6.9 Hz), 2.38 (s, 3H, CH_3_), 3.83 (s, 3H, OCH_3_), 4.20 (q, 2H, CH_2_, *J* = 6.9 Hz), 7.56–7.79 (m, 7H, Ar-H), 8.25 (s, 1H, =CH-Ar), 8.44 (s, 1H, 4-H), 12.40 (brs, 1H, NH); Anal. calcd. for C_24_H_21_N_3_O_5_S: C, 62.19; H, 4.57; N, 9.07; found: C, 62.18; H, 4.55; N, 9.09%.

*Synthesis of 3-chloro-3-(8-ethoxycoumarin-3-yl)acrylaldehyde* (**8**). A solution of compound **3** (10 mmol) in DMF (10 mL) was cooled in an ice bath, a 1:1 mixture of DMF (10 mmol) and (10 mmol) POCl_3_ was added to it dropwise while the temperature was kept at 0 °C. The resulting mixture was stirred for 2 hours at 0 °C, hen it was allowed to stir at room temperature for 3 hours more and poured onto crushed ice. The solid formed was filtered, washed with water and dried under vacuum. The crude product was recrystallized from ethanol to give pale yellow needles; yield 94%; m.p. 123–125 °C; IR (KBr) υ (cm^−1^): 3,094, 2,978, 2,928 (CH stretching), 1,720, 1,668 (CO); ^1^H-NMR (DMSO-d_6_) δ: 1.42 (t, 3H, CH_3_, *J* = 7.2 Hz), 4.21 (q, 2H, CH_2_, *J* = 7.2 Hz), 7.41 (d, 1H, vinyl proton, *J* = 6.9 Hz), 7.33–7.53 (m, 3H, Ar-H), 8.81 (s, 1H, H-4), 10.19 (d, 1H, CHO, *J* = 6.9 Hz); MS *m/z* (%): 280 (M^+^+2, 3.1), 278 (M^+^, 6.4), 252 (3), 250 (9), 224 (6), 222 (20), 187 (100); Anal. calcd. for C_14_H_11_ClO_4_: C, 60.34; H, 3.98; found: C, 60.43; H, 3.96%.

*Synthesis of 4-ethoxy-9-(8-ethoxycoumarin-3-yl)-7-hydroxy-6H-benzo[c]chromen-6-one* (**9**). *Method A*: A solution of **1** (10 mmol), malononitrile (10 mmol) in absolute ethanol (30 mL) was refluxed in the presence of TEA (0.2 mL). A solid formed after 30 min. boiling and the refluxing were continued for 1.5 hours more. The crude product was recrystallized from an ethanol/benzene mixture to give colourless crystals; yield 77%; m.p. 236–8 °C; IR (KBr) υ (cm^−1^): 3,425 (OH), 3,094, 2,978, 2,928 (CH stretching), 1,704, 1,694 (CO); ^1^H-NMR (DMSO-d_6_) δ: 1.45 (t, 3H, CH_3_, *J* = 7.0 Hz), 4.20 (q, 2H, CH_2_, *J* = 7.0 Hz), 7.30–8.16 (m, 8H, Ar-H), 8.55 (s, 1H, H-4) and 11.22 (s, 1H, enolic OH, exchangeable by D_2_O); MS *m/z* (%): 444 (M^+^, 68), 388 (100), 360 (28), 304 (21), 189 (15); Anal. calcd. for C_26_H_20_O_7_: C, 70.25; H, 4.50. Found: C, 70.27; H, 4.52%. *Method B*: The reaction was carried out as described in Method A, but using ethyl cyanoacetate (1.13 mL) instead of malononitrile; yield (82%) (identity confirmed by m.p. and mixed m.p.).

*Synthesis of 2-amino-4,6-bis(8-ethoxycoumarin-3-yl)benzonitrile* (**10**). The reaction was carried out with the same procedure described for compound **9** (Method A) but using absolute methanol as a solvent and piperidine as a catalyst; m.p. 330–332 °C, 75%; IR (KBr) υ (cm^−1^): 3,474, 3,320 (NH_2_), 3,092, 3,042, 2,980 (CH stretching); 2,206 (CN), 1,720, 1,696 (CO); ^1^H-NMR (DMSO-d_6_) δ: 1.43 (t, 3H, CH_3_, *J* = 7.2 Hz), 4.23 (q, 2H, CH_2_, *J* = 7.2 Hz), 7.31 (m, 10H, Ar-H+NH_2_), 7.69 (s, 1H, H-4), 8.37 (s, 1H, H-4); MS *m/z* (%): 494 (M^+^, 27), 412 (100), 384 (19), 344 (28), 316 (44), 243 (19), 242 (10), 206 (22); Anal. calcd. for C_29_H_22_N_2_O_6_: C, 70.44; H, 4.48; N, 5.67; found: C, 70.40; H, 4.47; N, 5.68%.

*Synthesis of 3-(quinoxalin-2-yl)-8-ethoxycoumarin hydrobromide* (**11**). A solution of **4** (5 mmol) and *o*-phenylenediamine (5 mmol) in absolute methanol (20 mL) was refluxed for 3 hours. The solid obtained was filtered, washed with ethanol and dried under vacuum. The crude product was recrystallized from ethanol/benzene mixture to give compound **11** as pale brown crystals; yield 77%; m.p. 208–209 °C; IR (KBr) υ (cm^−1^): 3,417 broad (NH), 3,098, 3,022, 2,987, 2,896 (CH stretching); 1,720 (CO); ^1^H-NMR (DMSO-d_6_) δ: 1.43 (t, 3H, CH_3_, *J* = 7.1 Hz), 4.21 (q, 2H, CH_2_, *J* = 7.1 Hz), 7.37–8.15 (m, 8H, Ar-H), 8.88 (s, 1H, H-4), 9.58 (brs, 1H, NH, exchangeable by D_2_O); MS *m/z* (%): 400 (M^+^+2, 75), 398 (M^+^, 76), 370 (98), 368 (100), 289 (69), 205 (96), 102 (53), 76 (57); Anal. calcd. for C_19_H_15_BrN_2_O_3_: C, 57.16; H, 3.79; N, 7.02; found: C, 57.26; H, 3.69; N, 6.98%.

*7-(Bromo-(8-ethoxycoumarin-4-yl)methylene)-4-ethoxychromeno[4,3-d]pyrido[1,2-a]-pyrimidin-6(7H)-one* (**12**). Compound **12** was prepared from **4** (5 mmol) and 2-aminopyridine (5 mmol) according to the procedure described for **11** to give **12** from acetic acid as pale brown crystals; yield 63%; m.p. 231–233 °C; IR (KBr) υ (cm^−1^): 2,924 (CH stretching), 1,713, 1,692 (CO); ^1^H-NMR (DMSO-d_6_) δ: 1.18 (t, 3H, CH_3_, *J* = 7.1 Hz), 1.37 (t, 3H, CH_3_, *J* = 6.9 Hz), 4.02 (q, 2H, CH_2_, *J* = 7.1 Hz), 4.18 (q, 2H, CH_2_, *J* = 6.9 Hz), 6.46 (s, 1H, H-3, coumarin), 7.04–8.36 (m, 10H, Ar-H, pyridine-H); MS *m/z* (%): 574 (M^+^+2, 25), 572 (M^+^, 31), 493 (100), 465 (97), 304 (49), 280 (30), 224 (51), 196 (41), 134 (17); Anal. calcd. for C_29_H_21_BrN_2_O_6_: C, 60.75; H, 3.69; N, 4.89; found: C, 60.85; H, 3.68; N, 4.91%.

*Synthesis of 2-bromomethylene-8-ethoxy-2H-chromene-3-carboxylic (methyl-carbonimidic)thioanhydride* (**16**). A solution of compound **4** (10 mmol) in absolute methanol (40 mL) was refluxed with potassium thiocyanate (10 mmol) for 2 hours. The solid formed on cooling filtered off, washed with ethanol and dried under vacuum. The product was then recrystallized from acetic acid to give **16** as brown needles; yield 83%; m.p. 165–167 °C; IR (KBr) υ (cm^−1^): 3141 (NH), 3093, 3021, 2985, 2890 (CH stretching); 1717 (CO); ^1^H-NMR (DMSO-d_6_) δ: 1.41 (t, 3H, CH_3_, *J* = 7.1 Hz), 4.10 (s, 3H, OCH_3_), 4.17 (q, 2H, CH_2_, *J* = 7.1 Hz), 7.16–7.54 (m, 4H, Ar-H, NH), 7.88 (s, 1H, =CH), 8.58 (s, 1H, H-4); MS *m/z* (%): 385 (M^+^+2, 37), 383 (M^+^, 42), 303 (100), 355 (37), 276 (67), 232 (44), 218 (28); Anal. calcd. for C_15_H_14_BrNO_4_S: C, 46.89; H, 3.67; N, 3.65; found: C, 47.01; H, 3.67; N, 3.67%.

*4-Bromo-2-(3-ethoxy-2-hyroxybenzylidine)-3-hydroxybut-3-enoic cyanic thioanhydride* (**18**). A solution of **16** (10 mmol) was boiled in glacial acetic acid/methanol mixture (1:1; 40 mL). HCl gas stream was bubbled into the hot solution for 2 hours. The reaction mixture was allowed to cool down and the solid formed was filtered off and recrystalized from methanol as yellow crystals; yield 89%; m.p. 235–237 °C; Anal. calcd. for C_14_H_12_BrNO_4_S (368.97): C, 45.53; H, 3.25; Br, 21.41; N, 3.79; S, 8.67; found: C, 45.55; H, 3.27; Br, 21.42; N, 3.81; S, 8.68%.

*Synthesis of 3-ethoxy-2-hydroxybenzaldehyde azine* (**19**). A solution of compound **16** (5 mmol) in absolute methanol (20 mL) was stirred at room temperature with 85% hydrazine hydrate (10 mmol) for 4 hours. The solid obtained filtered, washed with ethanol (five times, 10 mL each) and dried under reduced pressure. The product was recrystallized from acetic acid to give **19** as yellow crystals; yield 93%; m.p. 275–277 °C; IR (KBr) υ (cm^−1^): 3,302 (OH), 3,083, 3,025, 2,965, 2,870 (CH stretching), 1,562, 1,597 (C=N); ^1^H-NMR (DMSO-d_6_) δ: 1.34 (t, 3H, CH_3_, *J* = 7.0 Hz), 4.09 (q, 2H, CH_2_, *J* = 7.0 Hz), 6.87–7.35 (m, 6H, Ar-H), 9.20 (s, 1H, =CH), 12.10, 12.45 (s, 1H, OH); MS *m/z* (%): 328 (M^+^, 100), 164 (57), 136 (46), 121 (51), 80 (65); Anal. calcd. for C_18_H_20_N_2_O_4_: C, 65.84; H, 6.14; N, 8.53; found: C, 65.81; H, 6.10; N, 5.55%.

*Synthesis of 3-(2-aminothiazol-4-yl)-8-ethoxycoumarin* (**20a**). Compound **4** (10 mmol) and thiourea (10 mmol) were disolved in absolute methanol (40 mL). The reaction mixture was refluxed for 1 hour. A precipitate formed on cooling, which was collected by filtration, washed with ethanol and dried under vacuum. The crude product was recrystallized from ethanol to give pale yellow crystals; yield 94%; m.p. 200–202 °C; IR (KBr) υ (cm^−1^): 3,387, 3,302, 3,148 (NH_2_), 3,020, 2,983, 2,887 (CH stretching), 1,705 (CO); MS *m/z* (%): 288 (M^+^, 100), 261 (26), 203 (21), 134 (22), 89 (40); Anal. calcd. for C_14_H_12_N_2_O_3_S: C, 58.32; H, 4.20; N, 9.72; found: C, 58.31; H, 4.18; N, 9.73%.

*Synthesis of 3-(2-(phenylamino)thiazol-4-yl)-8-ethoxycoumarin* (**20b**). Compound **20b** was prepared from **4** (10 mmol) and phenylthiourea (10 mmol) according to the procedure described for **20a** to give **20b** from ethanol/benzene mixture as yellow crystals; yield 69%; m.p. 216–218 °C; IR (KBr) υ (cm^−1^): 3,302, 3,148 (NH), 3,083, 3,011, 2,955, 2,850 (CH stretching), 1,697 (CO); ^1^H-NMR (DMSO-d_6_) δ: 1.41 (t, 3H, CH_3_, *J* = 7.0 Hz), 4.13 (q, 2H, CH_2_, *J* = 7.0 Hz), 7.00–7.76 (m, 7H, Ar-H), 8.61 (s, 1H, H-4), 10.31 (s, 1H, HNPh); MS *m/z* (%): 364 (M^+^, 100), 279 (27), 207 (12), 150 (39), 77 (41); Anal. calcd. for C_20_H_16_N_2_O_3_S: C, 65.92; H, 4.43; N, 7.69; found: C, 65.95; H, 4.40; N, 7.71%.

*Synthesis of 2-(4-(8-ethoxycoumarin-3-yl)thiazol-2-yl)acetonitrile hydrobromide* (**20c**). Compound **20c** was prepared from **4** (10 mmol) and cyanothioacetamide (10 mmol) according to the procedure described for **20a** to give **20c** from benzene as yellow crystals; yield 96%; m.p. 195–197 °C; IR (KBr) υ (cm^−1^): 1,722 (CO), 3,021, 2,946, 2,890 (CH stretching), 2,262 (CN); ^1^H-NMR (DMSO-d_6_) δ: 1.43 (t, 3H, CH_3_, *J* = 7.1 Hz), 4.16 (q, 2H, CH_2_, *J* = 7.1 Hz), 4.55 (s, 2H, CH_2_), 7.29–7.32 (m, 3H, Ar-H), 8.42 (s, 1H, H-4); ^13^C (75 MHz) (DMSO-d_6_) δ: 14.05 (CH_3_-ester), 22.50 (CH_2_-), 61.92 (CH_2_-ester), 115.47 (C-7), 116.43 (CN), 118.95 (C-5), 122.15 (C-4a), 124.26 (C-5 thiazole), 126.91 (C-6), 127.51 (C-3), 142.11 (C-4 thiazole), 144.92 (C-4), 151.32 (C-8a), 154.54 (C-8), 158.65 (C-2 thiazole), 159.89 (CO-lactone); MS *m/z* (%): 394 (M^+^+2, 24.9), 392 (M^+^, 28.9), 390 364 (M^+^-CO, 39.5), 312 (M^+^-HBr, 54.4), 284 (M^+^-(HBr+CO), 100), 256 (43.6), 227 (18), 198 (13.7), 134 (11.2), 89 (27.1), 63 (23.1); Anal. calcd. for C_16_H_13_BrN_2_O_3_S: C, 48.87; H, 3.33; N, 7.12; found: C, 49.25; H, 2.84; N, 7.20%.

*Synthesis of 2-(4-(8-ethoxycoumarin-3-yl)thiazol-2-yl)-3-ethoxyacrylonitrile hydrobromide* (**21**). A solution of compound **20c** (5 mmol) was refluxed with triethyl orthoformate (5 mL) for 3 hours. The excess triethyl orthoformate removed under reduced pressure. The residual solid was treated with ethanol, filtered off, washed with ethanol (three times, 10 mL each) and dried under vacuum. The crude product was recrystallized from benzene/petroleum ether 40–60 °C mixture to give compound **21** as green crystals; yield 83%; m.p. 145–147 °C; IR (KBr) υ (cm^−1^): 3,027, 2,966, 2,876 (CH stretching), 2,222 (CN), 1,728 (CO); ^1^H-NMR (DMSO-d_6_) δ: 1.39 (t, 3H, CH_3_, *J* = 6.9 Hz), 1.42 (t, 3H, CH_3_, *J* = 7.2 Hz), 4.17 (q, 2H, CH_2_, *J* = 6.9 Hz), 4.45 (q, 2H, CH_2_, *J* = 7.2 Hz), 7.28–7.45 (m, 3H, Ar-H), 8.15 (s, 1H, =CH), 8.43 (s, 1H, 4-H); MS *m/z* (%): 450 (M^+^+2, 10.1), 448 (M^+^, 12.1), 368 (100), 340 (50.1), 312 (67), 227 (29.3), 134 (35.7), 89 (49.9); Anal. calcd. for C_19_H_17_BrN_2_O_4_S: C, 50.79; H, 3.81; N, 6.23; found: C, 51.14; H, 3.35; N, 6.30%.

*Synthesis of 2-(4-(8-ethoxycoumarin-3-yl)thiazol-2-yl)-3-(dimethylamino)acrylonitrile hydrobromide* (**22**). A mixture of **21** (2.5 mmol) and dimethylamine (2.5 mmol) in absolute methanol (30 mL) was refluxed for 6 hours. The resulting solid was filtered off, washed with ethanol and dried. The crude product was then crystallized from acetic acid to give compound **22** as brown crystals; yield 89%; m.p. 206–208 °C; IR (KBr) υ (cm^−1^): 3,012, 2,944, 2,927, 2,890, 2,874 (CH stretching), 2,191 (CN), 1,720 (CO); ^1^H-NMR (DMSO-d_6_) δ: 1.41 (t, 3H, CH_3_, *J* = 6.9 Hz), 3.28 (s, 6H, (CH_3_)_2_), 4.18 (q, 2H, CH_2_, *J* = 6.9 Hz), 7.22–7.59 (m, 3H, Ar-H), 7.89 (s, 1H, C=CHNMe_2_), 8.12 (s, 1H, 4-H); MS *m/z* (%); 449 (M^+^+2, 14.1), 447 (M^+^, 18), 367 (M^+^-HBr, 100), 334 (35.8), 230 (28.6), 170 (18.3), 132 (29.1), 89 (48.9); Anal. calcd. for C_19_H_18_BrN_3_O_3_S: C, 50.90; H, 4.05; N, 9.37; found: C, 51.24; H, 3.61; N, 9.46%.

*Synthesis of 3-(2-(coumarin-3-yl)thiazol-4-yl)-8-ethoxycoumarin hydrobromide* (**24**). A solution of the acetonitrile derivative **20c** (10 mmol) in absolute methanol (40 mL) was refluxed with salicyaldehyde (10 mmol) in the presence of piperidine for 2 hours. The solid formed was filtered off, washed with ethanol and dried under reduced pressure. The crude product was recrystallized from acetic acid to give compound **24** as pale yellow crystals; yield 91%; m.p. 265–7 °C; IR (KBr) υ (cm^−1^): 2,948, 2,927, 2,897, 2,874 (CH stretching), 1,720 (CO); ^1^H-NMR (DMSO-d_6_) δ: 1.43 (t, 3H, CH_3_, *J* = 7.0 Hz), 4.19 (q, 2H, CH_2_, * J* = 7.0 Hz), 7.31–8.00 (m, 7H, Ar-H), 8.54, 8.95 (s, 1H, H-4), 9.14 (s, 1H, thiazole-H); Anal. calcd. for C_23_H_16_BrNO_5_S: C, 55.34; H, 3.24; N, 2.81; found: C, 55.77; H, 2.85; N, 2.84%.

*Synthesis of 3-(2-(2-imino-6,8-diiodo-coumarin-3-yl)thiazol-4-yl)-8-ethoxycoumarine hydrobromide* (**25**).Compound **25** was prepared from **20c** (10 mmol) and 3,5-diiodosalicyaldehyde (10 mmol) according to the procedure described for **24** to give **25** from benzene as pale green crystals; yield 96%; m.p. 310–312 °C; IR (KBr) υ (cm^−1^): 2,938, 2,927, 2,898, 2,877 (CH stretching), 1,720 (CO); MS *m/z* (%): 749 (M^+^, 15.9), 669 (100), 668 (59.6), 641 (69), 613 (24), 585 (14), 321 (13), 190 (15), 134 (14), 89 (19). Anal. calcd. for C_23_H_14_BrI_2_NO_5_S: C, 36.83; H, 1.88; N, 1.87; found: C, 36.98; H, 1.63; N, 1.89%.

*Synthesis of 3-(2-(6-(dimethylamino)-2-iminocoumarin-3-yl)thiazol-4-yl)-8-ethoxycoumarin hydrobromide* (**26**). Compound **26** was prepared from **20c** (10 mmol) and 5-dimethylaminosalicyaldehyde (10 mmol) according to the procedure described for **24** to give **26** from benzene as brown crystals; yield: 90% m.p. 223–5 °C; IR (KBr) υ (cm^−1^): 3,449, 3,209, (NH), 3,015, 2,978, 2,921, 2,890, 2,873 (CH stretching), 1,597 (C=N), 1,720 (CO); ^1^H-NMR (DMSO-d_6_) δ: 1.43 (t, 3H, CH_3_, *J* = 7.1 Hz), 3.31 (s, 6H, 2CH_3_), 4.19 (q, 2H, CH_2_, *J* = 7.1 Hz), 6.63–7.67 (m, 7H, Ar-H and thiazole-H), 8.39 (s, 2H, H-4); Anal. calcd. for C_25_H_22_BrN_3_O_4_S: C, 55.56; H, 4.10; N, 7.78; found: C, 55.88; H, 3.74; N, 7.83%.

### 3.2. Reaction of **20c** with Aromatic Aldehydes

A solution of **20c** (5 mmol) in absolute methanol (40 mL) was refluxed with 4-methoxybenzaldehyde and/or 4-chlorobenzaldehyde (5 mmol) in the presence of piperidine for 1 hour. The solid formed was filtered off, washed with ethanol and dried under reduced pressure. The crude product was recrystallized from a suitable solvent to give **27a**,**b**. The physical and spectral data of compounds **27a**,**b** are as follows:

*3-(4-Methoxyphenyl)-2-(4-(8-ethoxycoumarin-3-yl)thiazol-2-yl)acrylonitrile hydrobromide* (**27a**). Yellow crystals; yield 91%; m.p. 215–7 °C; IR (KBr) υ (cm^−1^): 3,425, 3,139, 2,923 (NH and CH stretching), 2,207 (CN), 1,728 (CO); MS *m/z* (%): 510 [M^+^] (7.5), 508 (76.3), 430 (100), 265 (15), 216 (42), 134 (15), 89 (34); Anal. calcd. for C_24_H_19_BrN_2_O_4_S: C, 56.37; H, 3.74; N, 5.48; found: C, 56.71; H, 3.37; N, 5.53%.

*3-(4-Chlorophenyl)-2-(5-bromo-4-(8-ethoxycoumarin-3-yl)thiazol-2-yl)acrylonitrile hydrobromide* (**27b**). Yellow crystals; yield 86%; m.p. 196–7 °C; IR (KBr) υ (cm^−1^): 3,011, 3,332.8 and 3,224.8, 2,923 (NHBr and CH stretching), 2,214 (CN), 1,720 (CO); MS *m/z* (%): 516 (M^+^+4, 31.5), 514 (M^+^+2, 100), 512 (M^+^, 34.9), 433 (76.5), 295 (46), 215 (63), 125 (57), 89 (69); Anal. calcd. for C_23_H_16_BrClN_2_O_3_S: C, 53.56; H, 3.13; N, 5.43; found: C, 53.90; H, 2.71; N, 5.48%.

*Synthesis of 2-(2-(4-methoxyphenyl)hydrazono)-2-(4-(8-ethoxycoumarin-3-yl)thiazol-2-yl)acetonitrile hydrobromide* (**28**). *Method A*: To a solution of compound **20c** (10 mmol) in methanol (20 mL), a combination of 4-methoxyaniline (10 mmol), hydrochloric acid (5 mL) and sodium nitrite (10 mmol) in glacial acetic acid (20 mL) at 0 °C was added dropwise. The resulting mixture was stirred at 0 °C for 1 hour then the stirring was continued for 2 hours more at room temperature. The solid formed was filtered, washed with ethanol and dried under vacuum. The crude product was recrystallized from dioxane to give pale green crystals; yield 73%; m.p. 258–60 °C; IR (KBr) υ (cm^−1^): 3,157 (NH), 3,011, 2,883 (CH stretching), 2,214 (CN), 1,735.8 (CO); ^1^H-NMR (DMSO-d_6_) δ: 1.44 (t, 3H, CH_3_, *J* = 6.9 Hz), 3.76 (s, 3H, OCH_3_), 4.18 (q, 2H, CH_2_, *J* = 6.9 Hz), 6.96–7.47 (m, 7H, Ar-H), 8.39 (s, 1H, H-4), 11.93 (s, 1H, NH, cancelled by D_2_O); MS *m/z* (%): 526 (M^+^, 12), 445 (100). Anal. calcd. for C_23_H_19_BrN_4_O_4_S: C, 52.38; H, 3.63; N, 10.62; found: C, 52.69; H, 3.26; N, 10.69%. *Method B*: A solution of **4** (5 mmol) in absolute methanol (20 mL) was refluxed with (4-methoxyphenylazo)-2-cyanoethanethioamide **29** (5 mmol). The work up continued as previously mentioned in Method A to give **28**; yield 79 %; (identified by m.p. and mixed m.p.).

### 3.3. Antibacterial Activity

The newly synthesized compounds were screened for their antimicrobial activities *in vitro* against two Gram-negative *Bordetella bronchiseptica* (ATCC 4617) and *Escherichia coli* (ATCC 14169) and four Gram-positive *Bacillus pumilus* (ATCC 14884), *Bacillus subtilis* (ATCC 6633), *Staphylococcus aureus* (ATCC 29737) and *Staphylococcus epidermidis* (ATCC 12228) pathogenic bacteria and two fungi *Candida albicans* (ATCC 10231) and *Saccharomyces cervesia* (ATCC 9080). The activities of these compounds were tested using the disc diffusion method [40] for bacteria and the paper disk diffusion method [41] for fungi. The area of zone of inhibition was measured using Ampicillin (25 μg mL^−1^) as standard antibiotic Micostatin (25 μg mL^−1^) was used as a reference antifungal. The tested compounds were dissolved in *N,N*-dimethylformamide (DMF) to give a solution of 1 mg mL^−1^. The inhibition zones were measured in millimeters at the end of an incubation period of 48 hours at 28 °C. *N,N*-dimethylformamide (DMF) showed no inhibition zone. Test results are shown in Table 1.

**Table 1:** Antibacterial screening of the synthetic compounds.

Compd. No.^a^	Inhibition Zone Diameter in mm							
	*Bordetella bronchiseptica* ATCC 4617	*Escheri-chia coli* ATCC 14169	*Bacillus pumilus* ATCC 14884	*Bacillus Subtilis* ATCC 6633	*Staph.aureus* ATCC 29737	*Staph. Epidermidis* ATCC 12228	*Candida albicans* ATCC 10231	*Saccharomyces cerevisae* ATCC 9080
3	19	20	11	12	19	12	18	10
4	22	27	23	20	20	16	13	15
5	24	18	28	28	27	25	15	21
6	28	14	18	26	15	18	24	18
7	22	13	19	23	14	13	17	22
8	NA	NA	NA	NA	NA	NA	NA	NA
9	NA	13	NA	NA	10	NA	NA	NA
11	NA	NA	NA	NA	NA	NA	NA	NA
12	NA	14	NA	NA	14	NA	NA	NA
16	27	19	18	24	12	13	17	13
20a	NA	10	NA	NA	10	NA	NA	NA
20b	NA	11	NA	NA	12	NA	NA	NA
20c	13	10	18	11	11	20	14	19
21	22	10	18	20	11	19	22	11
22	20	11	20	19	13	9	13	15
24	13	10	NA	21	10	NA	NA	12
25	8	14	NA	14	15	NA	NA	14
27a	19	10	14	18	11	20	16	19
27b	20	10	14	21	11	13	19	11
28	27	10	22	22	11	19	23	20
Ampicillin*	24	25	20	25	26	25	-	-
Mycostatin*	-	-	-	-	-	-	22	24

NA = not active; Diameter of the hole = 10 mm; * 25 µg/mL, ^a^c = 1mg/mL of new compounds in DMF.

## 4. Conclusions

Our interest in the synthesis of the title compounds was to focus on their study as antimicrobial agents as a part of our program which is aimed at the development of new heterocyclic compounds as more potent antimicrobial agents. In this paper we reported the synthesis of some 8-ethoxycoumarin derivatives bearing side chains, thiazole derivatives and the antimicrobial evaluation of all the novel compounds. The structures of the novel compounds were elucidated on the basis of IR, ^1^H-NMR, ^13^C-NMR and MS data. The screening results demonstrated that replacing the hydrogen atom attached to the coumarin nucleus at C-3 with a side chain as in compound **5** and thiazoles **7** and **28** results in wide spectrum antimicrobial activity against all tested bacteria and fungi compared to ampicillin and mycostatin, while the other compounds with other side chains showed moderate to weak activity.
